# Acknowledgement to Reviewers of *Medical Sciences* in 2019

**DOI:** 10.3390/medsci8010003

**Published:** 2020-01-15

**Authors:** Medical Sciences Editorial Office

**Affiliations:** MDPI, St. Alban-Anlage 66, 4052 Basel, Switzerland

The editorial team greatly appreciates the reviewers who have dedicated their considerable time and expertise to the journal’s rigorous editorial process over the past 12 months, regardless of whether the papers are finally published or not. In 2019, a total of 105 papers were published in the journal, with a median time to first decision of 32.1 days and a median time from submission to publication of 56 days. The editors would like to express their sincere gratitude to the following reviewers for their generous contribution in 2019:
Aalkjær, ChristianLitofsky, Norman ScottAbrams, Cameron F.Liu, YingjunAbuzeid, Waleed M.Llanes, CarlosAdams, JamesLobmaier, SilviaAfolayan, Adeleye J.Long, YichengAgondi, RosanaLosurdo, GiuseppeAkamizu, TakashiLowres, NicoleAlbano, EmanueleMagit, AnthonyAmano, ToshiyasuMajor, Sofia O.Andrade, Vanda MariaMansson, ChristopherAngadi, SiddharthaMarrone, OresteAnsa, BenjaminMartinez, MercedesAntolak, HubertMazzilli, FernandoAntwi-Boasiako, CharlesMeerson, AriArciniega, Alejandra BenitezMehrotra, PrakharAuerbach, SanfordMeng, Yuan XiangBajaj, PuneetMichelle, PalmieriBaldwin, Paula KMielke, Howard W.Barcelos, AnabelaMilenkovic, Vladimir M.Basit, SulmanMiller, Anthony B.Batinic-Haberle, InesMiller, JoshuaBaudoin, TomislavMitchell, Cassie S.Bevilacqua, ArturoMo, FeiBidoli, EttoreMobley, David F.Bielsa, SilviaMonsuez, Jean-JacquesBitar, Mohamad AMorales-Sánchez, AbigailBlacher, EranMorgante, GiuseppeBlesson, Chellakkan SelvanesanMoskot, MartaBoels, Anne MeikeMou, LishaBoelsbjerg, Hanne BessMuñoz-López, FranciscoBonanno, ElenaMurray, Thomas ScotBonny, Andrea E.Musumeci, GiuseppeBorovac, Josip A.Nair, SreenathBosso, JohnNakai, YousukeBotelho, MonicaNakamura, TomokiBozic, JoskoNassini, RominaBrindley, DavidNeels, OliverBrown, Joshua DavidNemeș, Roxana MariaBuonerba, CarloNesterkina, MariiaCacoub, PatriceOdland, JonCadamuro, MassimilianoOhji, GohCantalupo, AnnaOren, MosheCantor, FredricOrzelska-Górka, JolantaCapoccia, MassimoOsier, NicoCardozo, Licy YanesPaal, PiretCarotenuto, MarcoPabbidi, Reddy MCarre, AurorePaine, AnantaChaigne, AgathePalombi, LeonardoChan, Ben C. L.Panda, Amaresh C.Chang, Hui-huaPapp-Wallace, Krisztina M.Chang, R. JeffreyPark, EugeneChang, Theresa Li-YunPark, MinsunChattopadhyay, SubhasisParrington, LucyChi, Yung-WeiPasquinelli, GianandreaChina, ArnabPaulsson, Johan O.Chuang, Hung-YiPejin, BorisCiciliot, StefanoPentaris, PanagiotisCingi, CemalPetkov, Georgi V.Coubally, JeanPhilpott, Carl M.Coussons, PeterPilon, MathieuCox, LauraPolich, GingerCramb, SusannaPrior, SarahCrisafulli, ErnestoProkopakis, EmmanuelCullen, NoraQuadri, SyedaCvorovic, JelenaQuallich, SusanneD’Andrea, SettimioQuartuccio, LucaDe Leeuw, PeterRallis, MichailDe Leo, VincenzoRamsay, PamelaDel Mistro, AnnarosaRao, FaizaDelfín, Dawn A.Rato, Luís Pedro FerreiraDiawara, Moussa M.Reeve, AmyDikalov, SergeyReznik, Jacqueline E.Dimopoulos, KonstantinosRimmer, JanetDionne, AudreyRipellino, PaoloDol, Kim SangRossi, ClaudiaDomingues, Renan B.Rupprecht, SilkeDrane, PatrickRusu, Alina SimonaElbasiouny, SherifRybak, Leonard P.Errichiello, EdoardoSamson, SureshEsteve-Puig, RosauraSamuels, BrianExbrayat, Jean-MarieSaraux, AlainFacio Jr, Fernando NestorSarkar, AmritaFalup-Pecurariu, OanaSaxena, VikasFedotova, Julia O.Schleicher, Erwin D.Ferdinand, Keith CopelinSchmid, Kurt WernerFernandez, Teresa TórtolaSchneider, JoséFernando III, Antonio T.Schrier, RachelFinco, IsabellaSchughart, KlausFischer, WolfgangShah, Rachana D.Florea, Ana-MariaShahi, ShaileshFontana, PaoloShahshahani, AmirhosseinFransen, PaulShapiro, Charles L.Frascoli, MichelaShea, KylaFraser, Matthew O.Shimada, MasakoGalli, FrancescoSiegel, Robert M.Gambineri, AlessandraSimone, DonaldGani, FedericaSîrbulescu, Ruxandra F.Garus-Pakowska, AnnaSohn, SeonghyangGiron, Maria CeciliaSomboonwit, CharurutGnanamony, ManuSong, Lu-JieGray, Peter B.Spassov, SashkoGrazette, LuandaSpiotto, Michael T.Greim, HelmutStaniszewska, AnnaGridnev, Vladimir I.Stanojevic, Dragana M.Gubin, DenisStevenson, DonaldGude, FranciscoSun, YingjieGuerriero, GiuliaTamba, Bogdan IonelGunaje, JayaramaTanabe, KatsuyukiHansen, Keith A.Tanini, MarcoHart, Nicolas H.Testa, UgoHe, BulangThanigaimani, ShivshankarHeather, NatashaThayabaranathan, TharshanahHehlgans, StephanieTonolo, GiancarloHennessey, James V.Toth, Balazs IstvanHering-Smith, KathleenTrentini, AlessandroHileman, Stanley MTroyanov, StéphanHirst, JenniferTsai, Ming-ShaoHoeger, KathyTudó, GriseldaHopp, RussellTulloh, RobertHopp, Russell JamesTuncer, ÜlküHorvatits, ThomasTurnbull, Matthew W.Hsia, Shih-MinTuvdendorj, DemidmaaHsieh, Tung-ChinTylee, Tracy S.Huber, Peter E.Van Boven, Job FMHudlikar, RasikaVan Dijk, Jitse P.Huerta, MiguelVashist, SandeepIbáñez Escribano, AlexandraVenkatesan, ThiagarajanIeni, AntonioVercellotti, GregoryIentile, RiccardoVespa, Paul M.Ikeda, YoukoVidetta, WalterIkemori, AtsukoVišnjić, AleksandarIvanenko, AnnaVisser, Adriaan PhJackson, William F.Vita, RobertoJanikowska, GrażynaWada, JunJung, Chan KwonWakino, ShuKadotani, HiroshiWarrander, LynneKarlsson, ErikWatanabe, EiichiKebede, Mihiretu M.Winneke, GerhardKeisaku, SatoWinter, Michiel M.Kido, Mizuho A.Witchel, Selma FeldmanKim, Han-sukWood, Andrew JamesKing, Thomas F.J.Wrześniok, DorotaKissinger, PattyWu, Alexander THKlatt, MaryannaWu, Hung-TsungKoivisto, AriWu, ShengKolarik, Andrew J.Wu, Yu-ChangKoşar, FilizXu, JianKosinsky, Robyn LauraXu, JunwangKotyla, Przemyslaw J.Yanagi, NatsuyoKraus, ArminYang, KunKrishnakumar, DevadasYang, ZhihongKumamoto, EiichiYehuda, ShoenfeldKunicki, MichałYilmaz, BahtiyarLamas, AlexandreYip, Bon HamLan, Yuan-TzuYong, TuckLatalski, MichalYu, Chia-LiLee, Kyung-YilZhang, GuanshiLevkovich, InbarZhang, YinfengLewis, Ghislaine L.Zheng, PanLi, ShitaoZhou, TianhaoLiao, Yi-JenZuhl, MicahLicari, Amelia


